# A New Method of Using Gold Foil

**Published:** 1855-10

**Authors:** 


### ARTICLE X.

### *A New Method of Using Gold Foil.* By Prof. Arthur.

Some few months since a short article on "sponge gold," written by me, was published in the "News Letter." It was simply intended for the purpose of doing my part in calling the attention of the profession to it as a desirable improvement, and to express my confidence in it as a reliable material for filling teeth. I did not pretend to go at any length into the subject, but made some simple general statements, promising to prepare a detailed account, at some future time, of methods of using it which had enabled me to employ it with success. I had very soon found, after beginning to use the "sponge gold," that it could not be relied upon if the directions by which it had been accompanied, when sold, were followed; and I was not at all surprised that disappointment in the expected result occurred as a consequence of its use in this way. Heavy demands upon my time, however, have prevented me from preparing such an article as I intended, and it will now be rendered unnecessary, I presume, by a publication in preparation by Messrs. Watts & Co., of which I have seen a part of the proof-sheets. I have, indeed, within a short time past, fallen upon a new field of experiment, and find that by using gold foil in a way somewhat differing from that in which it is ordinarily used, it can be worked precisely like "sponge" or "crystallized" gold.

I have never regarded gold foil, as ordinarily used, as a perfect article for the purpose intended. There are very few members of our profession, I presume, who are not aware of its defects. That it has answered the purpose of filling teeth better than any substance brought before the profession, until lately, I am well aware, but still it has always been my desire, as I know it has been that of many others in the profession, to find something free from its defects.

It is well known that no adhesion takes place between the layers of gold foil, as ordinarily used, and the retension of the whole mass of a filling depends upon the form of the cavity, or upon the lateral force it is made to exert upon the walls. The manner in which most good operators proceed to fill a cavity with foil, no matter how they may prepare it, is to begin at one side and condense the filling toward that side, making sure to allow some of the gold to extend above the mouth of the cavity, far enough to make it level with it after it is condensed at the surface and properly burnished.

Now, it is impossible, if the filling is well condensed, to add more gold directly to the surface. The layers of foil, as ordinarily used, will not adhere to that portion which is previously condensed. It becomes necessary, in such case, to remove the whole filling, or to cut away so much of it, if it be large enough, as to furnish a reliable hold for the additional gold. This is a defect in foil for which I have always been anxious to find a remedy.

I found, by experiment, some few years ago, that by using heavy numbers of gold, a result might be obtained approximating, at least, what was desired in this way. I proposed to employ No. 30 gold foil, with sharp pointed instruments, as I found that by forcing the single strip of heavy gold into contact with portions previously introduced into the cavity, a kind of union between the different portions might be effected. In this way I found myself enabled to add strip after strip of gold to a filling, until I had accomplished the object in view. I made use of this form of gold foil, in this way for nearly two years, exclusively, and only abandoned it in consequence of the difficulty of keeping myself supplied with suitable instruments, which it is impossible to have properly made by an instrument maker. The points required are necessarily very hard, and easily broken. Still I accomplished with this form of gold all I expected from it, and abandoned its use for the reason stated alone. It is not improbable that gold in this form may still be found desirable, if used as I am about to propose.

I again went back to gold foil as I had formerly used it, and employed it in this way until my attention was called to the article introduced to the profession under the name of "sponge gold." The first I used was made by White, of Utica, and it at once struck me as being a most desirable improvement, in many respects, upon gold foil. A specimen of Watts & Co's gold was then put into my hands by the inventor, and I found it free from the objection of great friability, one of the most defective features of White's preparation. It was a beautiful specimen of this material, and I have never since found any which in all respects satisfied me so well. I took hold of the new form of gold eagerly, and soon found that by using it (very differently, however, from the manner described) I could obtain better results in my operations than I had ever been able to reach before. I used the "sponge gold" with greater satisfaction, for more than a year, than any other material I had ever taken hold of. I have seen my operations repeatedly, and at long periods of time, after they have been performed, and I have never in a singe instance observed any change in its appearance or consistency, except when from unfavorable circumstances the operation was imperfectly performed. I feel no hesitation, therefore, in saying, that the confidence I have expressed in it, in the article alluded to, I still retain.

But "sponge gold" has unquestionably, with all its advantages, its own defects, which have interfered with and must prevent its general adoption. Its friability leads, in many cases, to more or less waste. As far as my experience goes, it is exceedingly difficult to work, if it gets at all wet during the performance of an operation, and it certainly consumes more time to perform reliable operations with it, in the great majority of cases, than gold foil. Still its advantages, with all these defects, as I have said, were so great in bringing about desirable results, that I have been willing to devote the additional time for the sake of making more perfect operations when completed. If these difficulties can be overcome, and an article found free from the defects of "sponge gold," which can be worked in the same way as this material, that is, if adhesion can be obtained between the portions placed in a cavity, a further advance unquestionably is made.

In making some experiments for the purpose of testing the relative solidity, when condensed in a cavity, of gold foil and "sponge gold," I found the foil may be made to work in precisely the same way as the sponge gold, and as perfect adhesion obtained between the different portions of gold put into a cavity as with this material. Nothing more is necessary than to cut the sheets of the ordinary numbers of foil into two or three pieces, roll up very loosely, and hold it in the flame of a spirit lamp until it reaches a red heat. It will at once be found to have become so adhesive that, with small and sharply serrated instruments, it may be made to adhere as readily as the best specimens of "sponge gold." It is necessary, however, when gold foil is used in this way, that it should be cut into small portions, for the very adhesiveness which it acquires when treated as I have proposed, prevents it from working well if an attempt is made to use it in the ordinary manner.

The instruments which I have found best adapted to the use of gold in this way, are small flattened pluggers, with two sharp points cut at the end, and made so hard that they will not turn. The gold may be picked up with such instruments, carried to the desired place, and condensed precisely as "sponge gold." It is well to go over each piece added, with a single sharp point.

Although I have been making use of gold foil in this way for only about a month, I am exceedingly gratified at the results I have obtained, and am urged to call attention to it.

It seems very strange that so desirable an improvement as this should have been in our hands for so long a time, and yet not discovered. I presume many operators have attempted to restore gold foil, which had become impaired, by reheating it. I did so myself years ago, but found that the very adhesiveness it acquired by this process, interfered seriously with its use in the ordinary way.

I have felt it necessary to make this long statement in order to protect myself against the charge of vacillation, as I have at various times earnestly advocated several different methods of filling teeth. My strong desire has always been to improve, for my own benefit and that of others, the methods of performing the operations of our profession. I always have been, and always shall be, ready to take hold of any new thing which promises advantages, and to test it as thoroughly as I am capable of doing. I have not changed my ground in relation to any of the forms of gold I have advised to be used. Each has certainly the advantages, which I attributed to them. I do not now take the ground that I have been mistaken in my expressed views of "sponge gold;" I still regard it as perfectly reliable, when properly used. But for ordinary use, I am convinced that gold in the form I am now recommending it, is a decided improvement upon any method of using gold hitherto known. Whether it will be regarded as superior to "sponge gold," in the hands of those who have successfully used this material, is a question which time and trial can alone decide.

I have no idea that gold will be used generally in the manner I now recommend. It is exceedingly difficult to induce men to change a course which they have successfully pursued for years, and the difficulties of which they have learned to encounter and overcome, for any new thing. And, even if they are disposed to make trial of a new process, it is often so imperfect a trial, that it is unsatisfactory. With regard to the matter in hand, simple as it is, I confidently say to every operator in the profession, that if fairly tried it will afford advantages in the use of gold foil of which few have dreamed.

I may say that the foil of different manufacturers present a remarkable difference in regard to this quality of adhesiveness. It is certainly advisable to try several kinds, if that in hand does not work well. The profession may rely upon the full truth of the statements I have made. I have never, in the course of practice of fifteen years, made use of any material for filling teeth which has so fully satisfied me as foil used in the manner I have described.—*Dental News Letter.*

